# Ghrelin receptor regulates adipose tissue inflammation in aging

**DOI:** 10.18632/aging.100888

**Published:** 2016-01-30

**Authors:** Ligen Lin, Jong Han Lee, Eric D. Buras, Kaijiang Yu, Ruitao Wang, C. Wayne Smith, Huaizhu Wu, David Sheikh-Hamad, Yuxiang Sun

**Affiliations:** ^1^ USDA/ARS Children's Nutrition Research Center, Department of Pediatrics, Baylor College of Medicine, Houston, TX 77030, USA; ^2^ State Key Laboratory of Quality Research in Chinese Medicine, Institute of Chinese Medical Sciences, University of Macau, Macao, China; ^3^ Department of Internal Medicine at University of Michigan Health System, Ann Arbor, MI 48109, USA; ^4^ Department of Intensive Care Unit, the Third Affiliated Hospital, Harbin Medical University, Harbin, 150081, China; ^5^ Department of Medicine, Baylor College of Medicine, Houston, TX 77030, USA; ^6^ Department of Molecular and Cellular Biology, Baylor College of Medicine, Houston, TX 77030, USA; ^7^ Huffington Center on Aging, Baylor College of Medicine, Houston, TX 77030, USA; ^8^ Department of Nutrition and Food Science, Texas A&M University, College Station, TX 77843, USA

**Keywords:** ghrelin, growth hormone secretagogue receptor (GHS-R), adipose tissue macrophages (ATMs), inflammation, peritoneal macrophages (PM), thermogenesis

## Abstract

Aging is commonly associated with low-grade adipose inflammation, which is closely linked to insulin resistance. Ghrelin is the only circulating orexigenic hormone which is known to increase obesity and insulin resistance. We previously reported that the expression of the ghrelin receptor, growth hormone secretagogue receptor (GHS-R), increases in adipose tissues during aging, and old *Ghsr^−/−^* mice exhibit a lean and insulin-sensitive phenotype. Macrophages are major mediators of adipose tissue inflammation, which consist of pro-inflammatory M1 and anti-inflammatory M2 subtypes. Here, we show that in aged mice, GHS-R ablation promotes macrophage phenotypical shift toward anti-inflammatory M2. Old *Ghsr^−/−^* mice have reduced macrophage infiltration, M1/M2 ratio, and pro-inflammatory cytokine expression in white and brown adipose tissues. We also found that peritoneal macrophages of old *Ghsr^−/−^* mice produce higher norepinephrine, which is in line with increased alternatively-activated M2 macrophages. Our data further reveal that GHS-R has cell-autonomous effects in macrophages, and GHS-R antagonist suppresses lipopolysaccharide (LPS)-induced inflammatory responses in macrophages. Collectively, our studies demonstrate that ghrelin signaling has an important role in macrophage polarization and adipose tissue inflammation during aging. GHS-R antagonists may serve as a novel and effective therapeutic option for age-associated adipose tissue inflammation and insulin resistance.

## INTRODUCTION

Aging is commonly accompanied by increased fat mass and chronic low-grade inflammation, thus the concurrences of obesity and insulin resistance are significantly greater in aging [1, 2]. Epidemiological studies show that the prevalence of insulin resistance and type 2 diabetes (T2D) is clearly higher in the elderly [3]. However, it is not clear which is the cause and which is the effect between obesity and aging; this makes prevention/treatment of age-associated adipose inflammation extremely challenging. Adipose tissues store excess lipids and secrete various cytokines, which are involved in the etiology of inflammation, insulin resistance, and T2D. However, fat mass peaks at middle-age and early old age, but declines substantially in advanced aging [4]. Hence, the increased incidence of insulin resistance and T2D in aging could not be fully explained by obesity. Moreover, when subjects of all ages were matched by body weights and obesity indices, the age-associated decline in insulin sensitivity were still evident [5]. Similarly, the magnitude of age-associated decrease in lean body mass and physical activity did not match the age-associated increase in insulin resistance [6]. These observations suggest that factors other than obesity may drive the onset of insulin resistance in aging.

Obesity is often accompanied by low-grade chronic inflammation in adipose tissues, and exhibiting elevated pro-inflammatory cytokines such as tumor necrosis factor-α (TNF-α), interleukin-1β (IL-1β), interleukin-6 (IL-6) and monocyte chemotactic protein-1 (MCP1). Emerging evidences suggest that adipose tissue macrophages (ATMs) are a major pathogenic factor for insulin resistance [7-10]. MCP1 recruits circulating monocytes into adipose tissues, where monocytes become ATMs. ATMs consist of two subsets: pro-inflammatory M1 and anti-inflammatory M2. M1-like macrophages, express both F4/80 and CD11c but not CD206, produce pro-inflammatory cytokines such as TNF-α, IL-1β and IL-6, and are associated with an obese and insulin-resistant state. M2-like macrophages, express F4/80 and CD206 but not CD11c, produce anti-inflammatory cytokines such as IL-10, arginase 1 (Arg1) and YM-1, and are associated with a lean and insulin-sensitive state and secrete norepinephrine [7, 11]. M1 ATMs release pro-inflammatory cytokines which impair adipocyte function by inhibiting insulin action in the tissues [11, 12]. Macrophages preferentially infiltrate intra-abdominal fat more than subcutaneous fat; thus, intra-abdominal adiposity more often leads to insulin resistance [12]. Macrophages undergo changes in response to micro-environmental stimuli. It has been shown that high-fat diet and obesity induce phenotypic switch of ATMs from M2 to M1, leading to insulin resistance [13].

It has been proposed that inflammation is the harbinger of aging. Macrophages have central roles not only in the inflammatory and immune responses, but also in the stress response [14]. Inflammation is commonly coupled with aging and is central to the aging process, a phenomenon described as ‘inflamm-aging’ [14-16]. Adipose tissues include adipocytes and stromal vascular cells, the later contain macrophages. There are conflicting reports on whether adipocytes or ATMs are a prime driver of adipose tissue inflammation. Some studies show that the ATMs of old mice shifts toward pro-inflammatory state showing an increased M1/M2 macrophage ratio [14-16], suggesting that macrophages are responsible for the inflammatory state of the aging adipose tissues. However, there is also report showing inflammatory responses are up-regulated in adipocytes but not in macrophage-containing stromal vascular cells in old animals, suggesting that adipocytes are responsible for the inflammatory state of the aging adipose tissues [17]. Little is known about the genes that regulate ATMs and adipose tissue inflammation during aging, and knowledge of the role of M2 macrophages in adipose tissues is even scarcer. Identification of factors regulating macrophage infiltration in adipose tissues and controlling macro-phage polarization are important questions in obesity research.

Ghrelin is the only known orexigenic hormone to increase appetite and promote obesity [18, 19]. We and others have reported that ghrelin's effects on GH release and appetite are mediated through its receptor, the Growth Hormone Secretagogue Receptor (GHS-R) [20-22]. Ghrelin is ubiquitously expressed, but the highest expression level is detected in the stomach and intestine [23]. On the other hand, expression of GHS-R is more restricted; the high levels of expression are detected in pituitary and brain and low levels of expression are in selective peripheral tissues. Very low levels of GHS-R expression have been detected in white adipose tissue (WAT) and brown adipose tissue (BAT) [22, 24, 25]. We showed that GHS-R is expressed in both adipocytes and ATMs of old mice [26]. The role of ghrelin/GHS-R signaling in systemic inflammation is controversial; both pro-inflammatory and anti-inflammatory effects have been reported. Although ghrelin has been shown to have anti-inflammatory effects [27-31], it has also been reported that ghrelin increases neutrophil chemotactic factor IL-8 production in GHS-R transgenic colonic epithelial cells [32] and that GHS-R prompts the development of experimental colitis [33]. Very limited literature is currently available regarding ghrelin signaling in adipose tissue inflammation. We have reported that GHS-R ablation suppresses pro-inflammatory cytokine expression in ATMs of mice fed high fructose corn syrup (HFCS), but has no significant effect on fat mass [26]. In addition, we found that GHS-R ablation prevents age-associated obesity and insulin resistance, at least in part, it is mediated through increased thermogenesis in BAT [25, 34]. To better understand the role of ghrelin signaling in the regulation of age-associated adipose tissue inflammation, in the current study we have characterized the cellular phenotypes of peritoneal macrophages (PM) and ATMs of young and old wild-type (WT) and *Ghsr^−/−^* mice, and investigated the direct effects of GHS-R in macrophages.

## RESULTS

### Ablation of GHS-R attenuates age-associated increase of pro-inflammatory peritoneal macrophages

**Figure 1:**
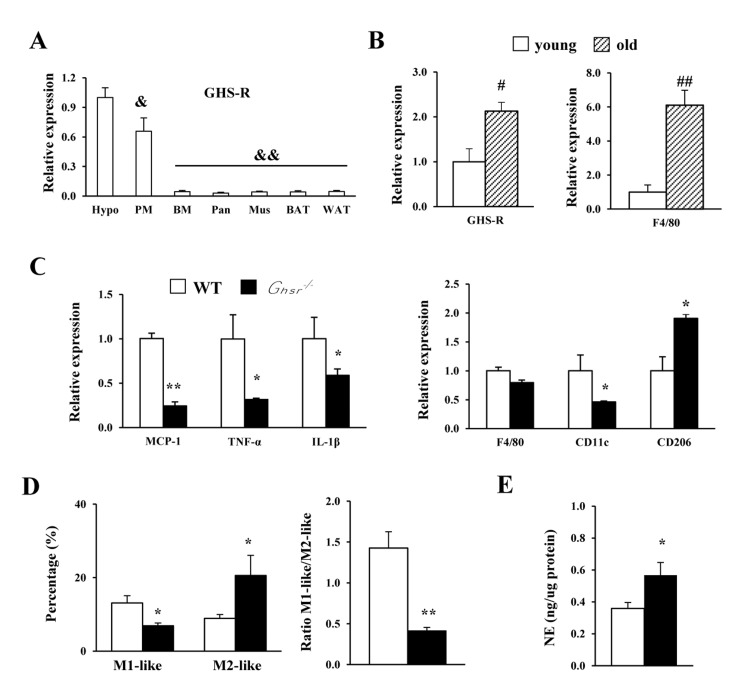
GHS-R ablation shifts peritoneal macrophages of old mice toward anti-inflammatory state, and releases more norepinephrine Young (4-5 months) and old (13-16 months) mice were used. (**A**) Expression of *Ghsr* gene in different tissues from WT mice. Hypo: hypothalamus; PM: peritoneal macrophages; BM: bone marrow; Pan: pancreas; Mus: skeletal muscle; BAT: brown adipose tissue; WAT: white adipose tissue. (**B**) Expression of *Ghsr* and *F4/80* genes in PM of young and old WT mice. (**C**) Expression of macrophage-related genes in PM of old WT and *Ghsr^−/−^* mice. (**D**) M1-like and M2-like macrophages, as well as ratio of M1-like/M2-like macrophages in PM of old WT and *Ghsr^−/−^* mice. (**E**) Norepinephrine (NE) levels in PM of old WT and *Ghsr^−/−^* mice. *N* = 6-10. &, *p*<0.05, &&, *p*<0.001, other tissue vs. hypothalamus; #*p*<0.05, ##*p*<0.001, old vs. young WT; *p<0.05, **p<0.001, WT vs. *Ghsr^−/−^*.

We previously demonstrated that GHS-R is expressed in ATMs [26]. Here, we compared GHS-R expression in peritoneal macrophages (PM) and non-elicited bone marrow (BM) of WT mice. Interestingly, GHS-R is relatively highly expressed in PM (60% of that in hypothalamus), when compared with non-elicited BM and peripheral tissues such as pancreas, muscle, BAT and WAT (Fig. 1A). We isolated PMs from young (4-5 months) and old (13-16 months) WT mice and found increased expression of GHS-R and macrophage marker gene *F4/80* with aging (Fig. 1B). Next, we assessed inflammatory status of PMs isolated from old WT and *Ghsr^−/−^* mice. In old *Ghsr^−/−^* mice, the expression of *MCP1*, *TNF-α* and *IL-1β* in PMs were greatly reduced, as compared to PMs of age-matched WT mice (Fig. 1C). While the macrophage marker *F4/80* was unchanged, the pro-inflammatory marker *CD11c* was lower and the anti-inflammatory marker *CD206* was higher in PMs of old *Ghsr^−/−^* mice, as compared to PMs of old WT mice (Fig. 1C). To further characterize the subtypes of macrophages, flow cytometry was employed to analyze the PMs. In agreement with gene expression data, we observed decreased M1-like macrophages (F4/80^+^:CD11c^+^:CD206^−^), increased M2-like macrophages (F4/80^+^:CD206^+^:CD11c^−^), and lower M1/M2 ratio in *Ghsr^−/−^* mice (Fig. 1D). Norepinephrine plays a critical role in macrophage proliferation, differentiation and function [35]. Norepinephrine has been shown to promote M2 macrophage activation [36].

Our previous studies showed that norepinephrine levels are higher in the urine of old *Ghsr^−/−^* mice [34]. In the current study, we found significantly higher norepinephrine levels in PMs of old *Ghsr^−/−^* mice compared with PMs of old WT mice (Fig. 1E). Together, these results suggest that GHS-R affects peritoneal macrophage polarization. GHS-R ablation has differential effects on M1 and M2 peritoneal macrophages in aging: reduced M1 and increased M2. Moreover, GHS-R deleted peritoneal macrophages exhibit characteristics of alternative activation that produces more norepinephrine.

### Ablation of GHS-R reduces age-associated inflammation in visceral WAT

**Figure 2:**
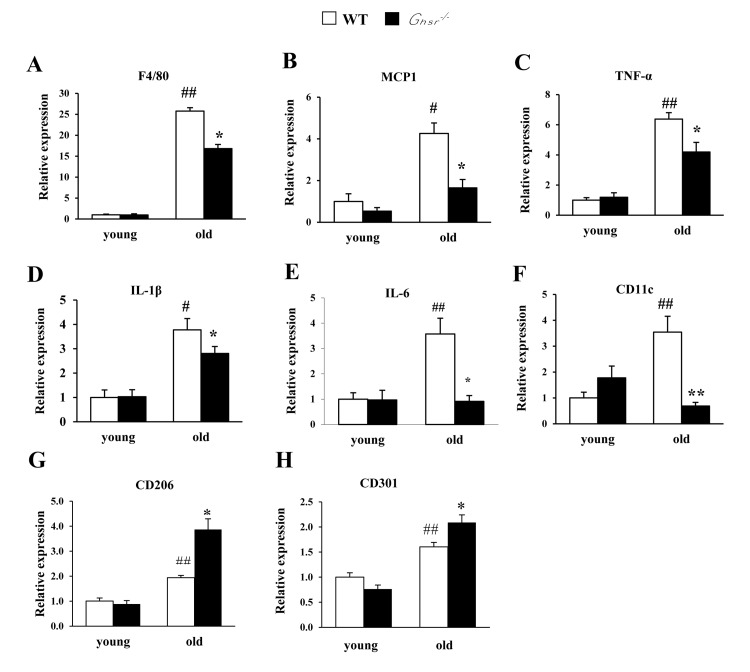
GHS-R ablation suppresses age-associated increase of expression of pro-inflammatory cytokines in WAT Young (4-5 months) and old (13-16 months) mice were used. Expression of *F4/80* (**A**), *MCP1* (**B**), *TNF-α* (**C**), *IL-1β* (**D**), *IL-6* (**E**), *CD11c* (**F**), *CD206* (**G**), and *CD301* (**H**) genes in epididymal WAT from young and old WT and *Ghsr^−/−^* mice. *N* = 6. #*p*<0.05, ##*p*<0.001, old vs. young WT; *p<0.05, **p<0.001, WT vs. *Ghsr^−/−^*.

Aging is associated with increased macrophage infiltration and higher production of pro-inflammatory cytokines in adipose tissues [11, 17]. To assess whether the lean and insulin-sensitive phenotype of old *Ghsr^−/−^* mice [25] is due to reduced macrophage infiltration and lower pro-inflammatory cytokine production in visceral WAT, the expression of *F4/80*, *MCP1*, pro-inflammatory cytokines (*TNF-α*, *IL-1β* and *IL-6*), and *CD11c* in epididymal WAT of young and old WT and *Ghsr^−/−^* mice were measured using real-time PCR. While the expression of *F4/80*, *MCP1*, TNF-α, IL-1β, *IL-6* and *CD11c* were unchanged in epididymal WAT of young *Ghsr^−/−^* mice, they were significantly reduced in epididymal WAT of old *Ghsr^−/−^* mice, (Fig. 2A-2F), indicating reduced inflammation in the visceral fat of old *Ghsr^−/−^* mice. These data are consistent with the improved insulin-sensitive phenotype we observed in old *Ghsr^−/−^* mice [25]. Next, we assessed the levels of anti-inflammatory M2 macrophage markers, including *CD206* and *CD301*, in epididymal WAT of young and old mice. We detected increased expression of *CD206* and *CD301* with aging; remarkably, the expression of these M2 gene markers was significantly up-regulated in old *Ghsr^−/−^* mice, but not in young mice (Fig. 2G and 2H). These results indicate that GHS-R ablation protects against age-associated inflammation of WAT.

### Ablation of GHS-R reduces age-associated pro-inflammatory macrophage infiltration in visceral WAT

**Figure 3:**
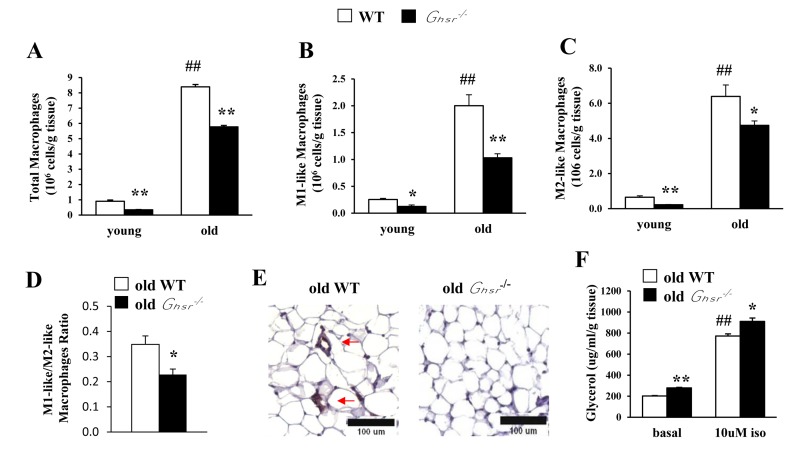
GHS-R ablation reduces macrophage infiltration into WAT, decreases pro-inflammatory M1-like macrophages and reduces M1/M2 macrophage ratio in WAT of old mice Young (4-5 months) and old (13-16 months) mice were used. Total macrophages (**A**), M1-like macrophages (**B**), M2-like macrophages (**C**), and the ratio of M1-like/M2-like macrophages (**D**) in epididymal WAT from old WT and *Ghsr^−/−^* mice. (**E**) Immunohistochemical staining of epididymal WAT from old WT and *Ghsr^−/−^* mice with anti-F4/80 antibody. Red arrows point to the “crown-like structures” macrophages. (**F**) *Ex vivo* lipolysis of WAT of old WT and *Ghsr^−/−^* mice, treated with or without 10 μM isoproterenol. *N* = 6-10. ##*p*<0.001, old vs. young WT; *p<0.05, **p<0.001, WT vs. *Ghsr^−/−^*.

Adipose tissue inflammation might be due to changes in absolute cell population of M1 or M2 macrophages and/or the ratio of M1/M2 subtypes. Thus, we used flow cytometry to assess the cell numbers of different subtypes of macrophages in epididymal WAT of young and old WT and *Ghsr^−/−^* mice. Total ATMs as well as individual M1-like and M2-like macrophages were significantly increased with age (Fig. 3A-3C). GHS-R ablation resulted in lower total ATMs as well as reduced M1-like and M2-like macrophages in both young and old mice (Fig. 3A-3C), which suggests reduced macrophage infiltration into adipose tissues.

High-fat diet-induced obesity has been shown to be associated with increased ratio of M1/M2 macrophages [7]. Similarly, we detected decreased ratio of M1-like:M2-like macrophages in old *Ghsr^−/−^* mice (Fig. 3D), suggesting reduced infiltration of M1 macrophages. To further confirm the finding, immunohistochemistry of F4/80 was conducted. As expected, the hallmark “crown-like” structure of M1 macrophages was detected only in sections of epididymal WAT from old WT mice, but not in that of old *Ghsr^−/−^* mice (Fig. 3E). These data lend support the notion that GHS-R ablation reduces M1 macrophage infiltration into adipose tissues, and promotes ATM polarization toward an anti-inflammatory M2 state in WAT during aging. Thus, GHS-R may serve as a crucial mediator of age-associated adipose inflammation.

### GHS-R ablation promotes *ex vivo* lipolysis of WAT from old mice

M2 macrophages use fatty acid oxidation to fuel mitochondrial oxidation; thus, lipolysis plays a key role in activation and function of M2 macrophages [37]. To elucidate the metabolic outcome of increased M2 macrophages, we studied *ex vivo* lipolysis of epididymal WAT from old WT and *Ghsr^−/−^* mice. Both basal and isoproterenol-stimulated lipolysis were increased in epididymal WAT of old *Ghsr^−/−^* mice (Fig. 3F). The data suggest that GHS-R ablation increases lipolysis of WAT, which is in line with our findings showing that macrophages of old *Ghsr^−/−^* mice favor alternative activation.

### Ablation of GHS-R decreases age-associated inflammation in BAT

**Figure 4:**
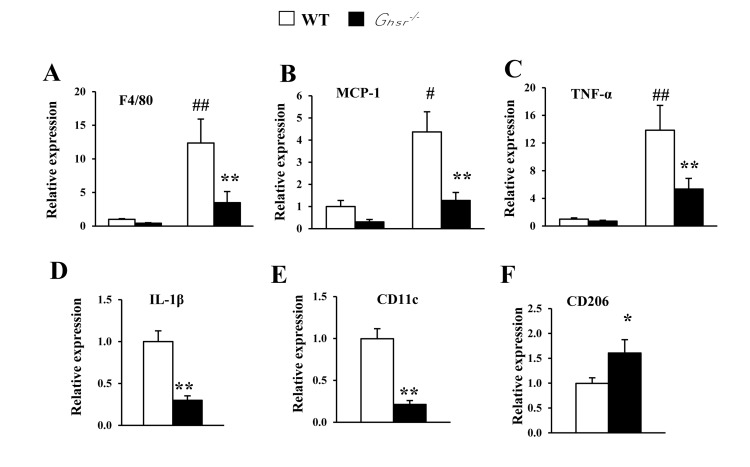
GHS-R ablation decreases age-associated increases of inflammation in BAT Young (4-5 months) and old (13-16 months) mice were used. Expression of *F4/80* (**A**), *MCP1* (**B**) and *TNF-α* (**C**) genes in BAT from young and old WT and *Ghsr^−/−^* mice. Expression of *IL-1β* (**D**), *CD11c* (**E**) and *CD206* (**F**) genes in BAT of old WT and *Ghsr^−/−^* mice. *N* = 6-8. #*p*<0.05, ##*p*<0.001, old vs. young WT; *p<0.05, **p<0.001, WT vs. *Ghsr^−/−^*.

Anti-inflammatory cytokine IL-4 has been shown to promote alternative activation of macrophages, which simulates thermogenesis [38]. In the current experiment, we assessed the impact of GHS-R ablation on cytokine profiles in BAT. The expression of pro-inflammatory *F4/80*, *MCP1*, *TNF-α*, *IL-1β* genes were increased in BAT of old WT mice (Fig. 4A-4D). Interestingly, the expression of these pro-inflammatory cytokines was markedly reduced in BAT of old *Ghsr^−/−^* mice (Fig. 4A-4D). Furthermore, in BAT of old *Ghsr^−/−^* mice, we found that expression of the M1 macrophage marker *CD11c* was decreased (Fig. 4E), while the expression of M2 macrophage marker *CD206* was increased (Fig. 4F). These data suggest that GHS-R ablation mitigates age-associated inflammation in BAT and promotes macrophage polarization toward M2.

### GHS-R regulates pro-inflammatory cytokine release in RAW264.7 cells

**Figure 5:**
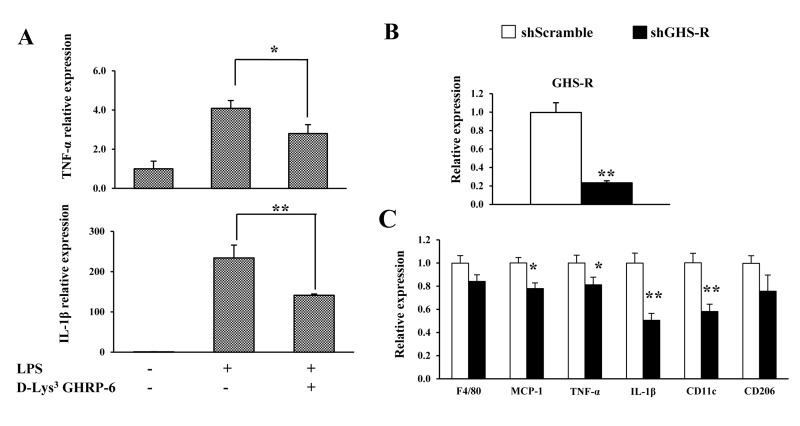
GHS-R affects inflammatory responses in macrophages *in vitro* (**A**) GHS-R antagonist suppressed *TNF-α* and *IL-1β* gene expression in LPS (1 μg/ml)-treated RAW264.7 cells with or without GHS-R antagonist D-Lys^3^ GHRP-6 (10 μM). *N* = 6. *p<0.05, **p<0.001, LPS + Lys^3^ GHRP-6 vs. LPS control. (**B**) Expression of *Ghsr* in shGHS-R knockdown RAW264.7 cells. (**C**) Expression of *F4/80*, *MCP1*, *TNF-α*, *IL-1β*, *CD11c* and *CD206* in shGHS-R knockdown RAW264.7 cells. shScramble represents scramble shRNA; shGHS-R represents GHS-R specific shRNA. *N* = 9. *p<0.05, **p<0.001, shScramble vs. shGHS-R.

In this set of experiments, we sought to determine the role of GHS-R in LPS-induced inflammation in cultured murine macrophages (RAW264.7 cells). Suppression of GHS-R expression in macrophages was achieved using GHS-R antagonist (D-Lys^3^ GHRP-6) or shGHS-R. LPS treatment robustly increased *TNF-α* and *IL-1β* expression, while 10 μM GHS-R antagonist protected against LPS-induced pro-inflammatory cytokine expression (Fig. 5A). The shGHS-R yielded an 80% reduction of GHS-R expression compared with scrambled shScr-treated cells (control) (Fig. 5B). Pre-treatment with shGHS-R reduced LPS-induced expression of pro-inflammatory markers *MCP1*, *TNF-α* and *IL-1β*, without affecting the expression of macrophage marker *F4/80* (Fig. 5C). In addition, we observed that the expression of M1 macrophage marker *CD11c* was reduced, while expression of M2 macrophage marker *CD206* was unchanged (Fig. 5C). These data suggest that GHS-R has direct effects on the inflammatory responses of macrophages, and a GHS-R antagonist mitigates LPS-induced macrophage inflammation.

## DISCUSSION

The hallmark function of adipose tissue is to store excess lipids in the body, and supply free fatty acids as energy. Adipose tissue also serves as a major endocrine organ, secreting various hormones and cytokines which play crucial roles in normal metabolism and obesity-associated dysfunctions [39, 40]. The ‘Inflamm-aging’ theory suggests that increased inflammation is the central underlying mechanism of the aging process [14]. There is a clear correlation between adipose inflammation and the incidences of insulin resistance and T2D in aging [17]. Indeed, in current study, macrophage numbers and pro-inflammatory cytokine expression in intra-abdominal WAT were increased with aging. Remarkably, GHS-R ablation decreases pro-inflammatory gene expression in peritoneal macrophages, ATMs in both WAT and BAT, and promotes a macrophage shift toward an anti-inflammatory M2 phenotype.

Macrophages are a major source of inflammatory mediators in the body, and increased macrophage infiltration in adipose tissue has been shown to positively correlate with age-associated metabolic complications, neurodegenerative diseases and cardiovascular diseases [14, 17, 41]. Previous observations from our lab suggested that GHS-R ablation in mice leads to a lean, insulin-sensitive phenotype associated with increased thermogenesis in BAT [25, 34]. Our current data show that GHS-R ablation protects against age-associated inflammation in both WAT and BAT by reducing microphage infiltration and promoting anti-inflammatory macrophage polarization. The GHS-R deleted macrophages exhibit characteristics of alternative activation, producing higher norepinephrine.

There are some limitations with our study. Our current study was carried in GHS-R global null mice, thus we cannot exclude the possibility that the inflammation phenotype observed in our mice might be a secondary effect of obesity. It is well documented that obesity can lead to adipose tissue inflammation and increased pro-inflammatory cytokines [7, 8, 42]. However, it has also been reported that increased fat mass is not always correlated with increased inflammation [43, 44]. We have previously reported that HFCS-feeding induces severe adipose inflammation, but has no effect on obesity; interestingly, GHS-R ablation attenuates HFCS-induced insulin resistance [26]. Moreover, our current data showed that GHS-R is expressed in macrophages, and GHS-R knockdown in macrophage RAW264.7 cells directly decreases pro-inflammatory cytokines expression of TNF-α, IL-1β, MCP-1 and IL-6. While, we cannot exclude the possibility that the inflammation phenotype of GHS-R null mice might be attributable to obesity; our *in vitro* data do suggest that GHS-R has cell-autonomous effects in macrophages. Macrophage-specific deletion of GHS-R would be an ideal approach to further decipher whether GHS-R mediated adipose inflammation is dependent or independent of obesity.

Adiposity and insulin resistance in adipose tissues are pathognomonic to obesity [7, 8, 10]. Low-grade inflammation in the expanding adipose tissues is mediated by activation and recruitment of macrophages. The majority of adipose tissue-derived cytokines (TNFα, IL-6 and IL-1β) originate from non-fat cells, which primarily are produced by infiltrating macrophages [45-48]. M1 ATMs have been reported to increase during aging, with a concomitant decrease in M2 ATMs [49]. Our current data reveal an increase in total ATMs during aging, and GHS-R ablation reduces total macrophage content of both M1-like and M2-like macrophages; more importantly, GHS-R ablation decreases the ratio of M1-like:M2-like macrophages. These characteristics are indicative that GHS-R ablation promotes ATM anti-inflammatory phenotypic shift toward M2, which likely contributes to the lean and insulin-sensitive metabolic phenotype of old *Ghsr^−/−^* mice. Previous studies from our lab revealed that GHS-R ablation alleviates HFCS-induced adipose inflammation independent of fat mass [26]. Our current data show that GHS-R ablation decreases macrophage-mediated adipose tissue inflammation during normal aging. Thus, GHS-R signaling plays an important role in macrophage polarization. These new data underscore that GHS-R is an important regulator of macrophage polarization, and GHS-R ablation promotes an anti-inflammatory phenotypic shift of macrophages under both diet-induced and age-associated adipose inflammation.

Adipokines and cytokines, secreted by adipose tissue, are involved in a variety of physiologic and pathologic processes. MCP1, synthesized and secreted in many cell types, is important for macrophage recruitment into atherosclerotic tissue and chronic inflammatory lesions [50, 51]. MCP1 plays a central role in inducing insulin resistance in adipocytes and skeletal muscle [52]. In mice and humans, MCP1 production increases in plasma and adipose tissue in both diet-induced and genetically-induced obesity, and MCP1 promotes the recruitment of monocytes into the expanding adipose tissues [52, 53]. MCP1 has been shown to mediate macrophage infiltration into various central and peripheral tissues [54, 55]. Activator protein-1 (AP-1) and nuclear factor-kB (NF-kB) are known to function as transcriptional factors binding to the promoter region of MCP1 gene and regulate MCP1 gene expression [56, 57]. Protein kinase C (PKC) and protein tyrosine kinase (PTK) have been shown to be involved in the regulation of MCP1 expression [58]. Under obese condition, free fatty acids and pro-inflammatory cytokines induce MCP1 [54, 59, 60]. Over-nutrition or obesity activates the innate immune system by recruiting immune cells (such as macrophages and T cells) into adipose tissue, muscle, and liver. The infiltrated immune cells produce pro-inflammatory cytokines in the tissues and in the circulation, which promote low-grade chronic inflammation, eventually lead to the development of systemic insulin resistance [54]. Indeed, we found that MCP1 levels were increased in adipose tissue during aging. MCP1 was significantly suppressed in adipose tissue of old *Ghsr^−/−^* mice, which potentially explains the lower macrophage content in adipose tissues of old *Ghsr^−/−^* mice. Further studies are needed to further investigate the role of ghrelin signaling in MCP1 regulation.

Unbalanced production of pro-inflammatory and anti-inflammatory adipokines in WAT contributes to the development of metabolic syndrome [61]. Emerging evidence indicates that aging is associated with a state of chronic, low-grade inflammation. Excessive macrophage infiltration into adipose tissues is responsible for increased production of pro-inflammatory adipokines during the progression of chronic inflammation. The dynamic change found in the adipose tissue is considered “adipose tissue remodeling”, in which stromal cells change dramatically in number and cell type during the course of aging [39, 62]. Infiltration of macrophages into the adipose tissue precedes the development of insulin resistance in animal models, suggesting that macrophages are crucial for obesity-related adipose tissue inflammation. The new findings in this paper demonstrate that the ghrelin signaling pathway plays a key role in age-associated adipose tissue inflammation by regulating macrophage polarization.

Our current study shows that old *Ghsr^−/−^* mice have a higher percentage of M2-like macrophages, and PMs produce a higher level of norepinephrine. Alternatively-activated macrophages have an important role in lipid metabolism; M2 macrophages have been shown to secrete norepinephrine to increase lipolysis in WAT and thermogenesis in BAT [38]. Adipose tissue lipolysis and lipid uptake are critical for activation of M2 macrophages [37]. Our data reveal increased lipolysis in old *Ghsr^−/−^* mice under both basal and β3-adrenergic agonist-stimulated conditions. The results suggest that GHS-R ablation promotes free fatty acid release from WAT, which promotes lipid mobilization and provides more fuel sources for thermogenesis. We have previously shown that GHS-R ablation increases thermogenesis in BAT in aging [34]. Peritoneal macrophages of old *Ghsr^−/−^* mice produce higher norepinephrine, which is indicative of increased alternatively-activated M2 macrophage population. GHS-R deletion may lead to alternative activation of macrophages, which may contribute to the increased lipolysis in WAT and thermogenesis in BAT of old *Ghsr^−/−^* mice, ultimately leading to the lean and insulin-sensitive phenotype of old *Ghsr^−/−^* mice. GHS-R mediated adipose inflammation and NE production may be mediated by the direct effects of GHS-R in macrophages. It would be very informative to study macrophage phenotypic switch and NE levels in macrophages of macrophage-specific GHS-R knockout mice.

Lastly, our studies suggest that GHS-R has pro-inflammatory effects in aging, which is contrary to the notion that ghrelin has anti-inflammatory effects [28-30]. It is likely that different signaling cascades mediate the inflammatory effects of ghrelin and GHS-R. Ghrelin's effect on macrophages may not be mediated by GHS-R, or there might be other unidentified ligand activators of GHS-R. Our data suggest that GHS-R has direct effects on macrophages; macrophage-specific deletion of GHS-R in a mouse model would be beneficial for further deciphering the functional discrepancy between ghrelin and GHS-R in macrophages.

**Figure 6:**
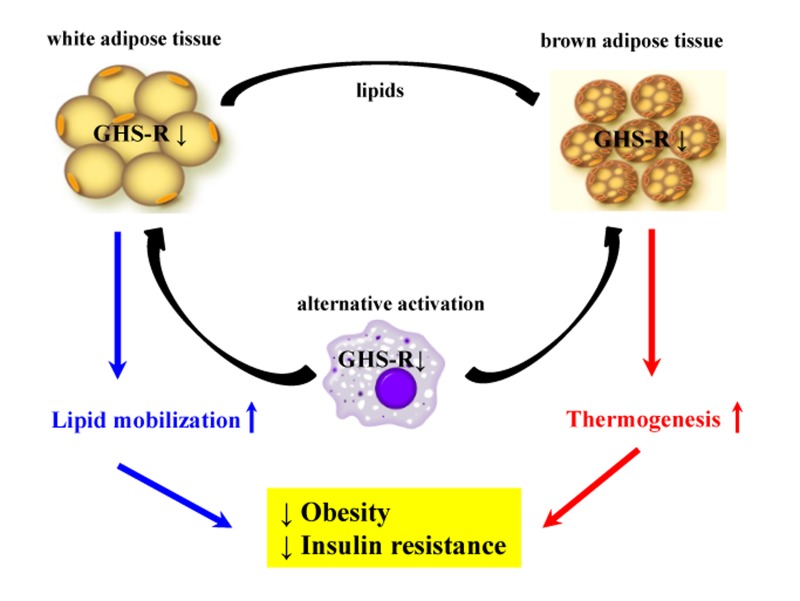
Schematic diagram of proposed role of GHS-R in adipose tissues during aging Our data demonstrate that GHS-R is an important regulator of macrophage polarization, and GHS-R has a pivotal role in adipose tissue inflammation in aging. GHS-R ablation shifts macrophage phenotype toward anti-inflammatory state and releases more norepinephrine. Alternatively-activated macrophages induced by GHS-R deletion infiltrate into adipose tissues, increasing lipid mobilization in WAT and activating thermogenesis in BAT, ultimately promoting a lean and insulin-sensitive metabolic state.

In summary, our studies suggest that GHS-R has very important roles in macrophage polarization. GHS-R is a key regulator of age-associated adipose tissue inflammation in both white and brown adipose tissues. GHS-R ablation shifts macrophages toward an anti-inflammatory state and leads to higher norepinephrine production in macrophages subsequently promoting lipid mobilization in WAT and thermogenesis in BAT (Fig. 6). Thus, GHS-R is an important regulator of macrophage polarization and GHS-R antagonists may serve as a unique class of anti-obesity drugs that can prevent/treat age-associated obesity and insulin resistance by suppressing adipose tissue inflammation, promoting lipid mobilization, and stimulating thermogenesis.

## METHODS

### Animals

All mice used in the experiments are congenic (backcrossed 13 generations to C57BL/6J background) male mice. We studied 4-5 month-old young and 13-16 month- old male mice. *Ghsr^−/−^* mice were generated as we previously described [20]. Wild-type (WT) and homozygous knockout mice (*Ghsr^−/−^*) were housed and bred in a pathogen-free facility at Baylor College of Medicine. Animals were housed under controlled temperature (75±1°F) and 12h light-dark cycles, with free access to food and water. Regular chow diet (2920X, 16% of calories from fat, 60% from carbohydrates, and 24% from protein) was from Harlan-Teklad (Indianapolis, IN). All experiments were approved by the Animal Care Research Committee of the Baylor College of Medicine.

### Real-time RT-PCR

Total RNA of WAT was isolated using TRIzol Reagent (Invitrogen, Carlsbad, CA), following the manufacturer's instructions. In order to remove potential genomic DNA contamination, RNA was treated with DNAse (Ambion, Carlsbad, CA) and run on gels to validate the purity and quality of the RNA. The cDNA was synthesized from 1μg RNA using the SuperScript III First-Strand Synthesis System for RT-PCR (Invitrogen, Carlsbad, CA). Real-time PCR (RT-PCR) was performed on an ABI 7900 using the SYBR Green PCR Master Mix, Taqman gene expression Master Mix (Applied Biosystems, Carlsbad, CA), or Bio-Rad using iQ SYBR GREEN supermix (Bio-Rad, Hercules, CA), according to the protocols provided by the manufacturers. 18s RNA and β-actin were used as internal controls. All primer and probe information are available upon request. Ghsr-1a primers used: forward 5′-GGACCAGAACCACAAACAGACA-3′, reverse 5′-CAGCAGAGGATGAAAGCAAACA-3′ [24]. This primer set flanks the intron, which allows us to distinguish its expression from GHS-R1b. Information for other primers is available upon request.

### PM isolation

Peritoneal macrophages were isolated as described previously [63]. Briefly, C57BL/6J mice were euthanatized by rapid cervical dislocation after anesthetization with isoflurane. Then, 5 ml ice-cooled PBS was injected into abdominal cavity. After gentle shaking for 3 minutes, abdominal fluid was collected into tubes using a syringe with 18G needle. Peritoneal macrophages were then collected by centrifugation at 2000 rpm (400g) for 10 minutes.

### Determination of norepinephrine level in PMs

The norepinephrine concentration in PMs was determined as previously described [38]. Briefly, PMs were homogenized by sonication in homogenization buffer (1 N HCl, 0.25 M EDTA, 1 mM Na_2_S_2_O_5_). After homogenization, cell debris was then removed by centrifugation at 13,000 rpm for 15 min at 4°C. The clear homogenate was collected and stored at −80°C for quantification. 50 μl homogenate was used for measurement of norepinephrine using ELISA assay (Rocky Mountain Diagnostics, Colorado Springs, CO) following the manufacturer's protocol. All samples were normalized to total protein content.

### Immunohistochemistry staining of WAT

For detection of F4/80, 5 μm paraffin-imbedded WAT sections were fixed and processed for immunohistochemistry using the avidin-biotin peroxidase complex (ABC, ThermoFisher, Waltham, MA) method and diaminobenzidine (DAB)-nickel reactions (Abcam, Cambridge, UK) as previously described [64]. Briefly, sections were de-paraffinized and dehydrated. Sections were incubated sequentially with 3% H_2_O_2_ to block peroxidase, F4/80 antibody (AbD SeroTec, Kidlinton, UK), then followed by secondary antibody (anti-rat) and ABC solution. After the DAB-nickel reaction, the sections were counterstained with 0.1% neutral red solution, and analyzed using a light microscope (Olympus DX 51, Olympus, Tokyo) and a charge-coupled device camera (Olympus DP12).

### Stromal vascular fraction isolation and flow cytometry analysis

Stromal vascular fraction was isolated as described previously [65, 66]. Briefly, 1g of epididymal adipose tissue was dissected and minced in Krebs-Ringer bicarbonate buffer (KRB) containing 1 mg/ml collagenase Type I (Worthington Chemicals, Lakewood, NJ). The solution was incubated in 37°C water bath for 30 minutes. The tissue slurry was then filtered through nylon mesh to remove undigested tissue, and centrifuged at 2200 rpm to fractionate adipocytes and stromal vascular fraction (SVF). SVF cells (1 × 10^6^ in a volume of 100 μl of PBS) were incubated with antibodies for flow cytometry analysis. The antibodies included PE anti-mouse F4/80 antigen (eBioscience, San Diego, CA), FITC anti-mouse CD11c antigen (BD Bioscience, San Jose, CA), purified CD16/CD32 antigen (BD Bioscience, San Jose, CA), and APC anti-mouse CD206 antigen (BD Bioscience, San Jose, CA). Cells were incubated with nonspecific IgG to assess background fluorescence (BD Bioscience, San Jose, CA). The data were collected using a FACScan and analyzed using CellQuest software (BD Biosciences, San Jose, CA).

### Cell culture

RAW264.7 cells were cultured in RPMI-1640 medium containing 10% bovine calf serum. 5 × 10^5^ RAW264.7 cells were seeded per well of a 6-well plate. 24 hours later, the cells were treated with 10 μM GHS-R antagonist D-Lys^3^ GHRP-6 (Sigma-Aldrich, St. Louis, MO), or vehicle. 4 hours later, the cells were treated with 1 μg/ml LPS (Sigma-Aldrich) for 16 additional hours followed by harvest.

### Generation of GHS-R knockdown RAW264.7 cell line and LPS treatment

RAW264.7 cells were cultured in RPMI1640 medium containing 10% bovine calf serum. GHS-R shRNA and scrambled shRNA were from Origene (Rockville, MD). The shRNAs were transfected into RAW264.7 cells using Lipofectamine® 2000™ (Invitrogen, Carlsbad, CA), according to the manufacturer's instructions. Briefly, 1 million RAW264.7 cells were seeded onto a 10 cm dish for 12 hours. Two hours before transfection, the medium was changed. 5 μg shRNA plasmids were mixed with Lipofectamine® 2000™ reagent, kept at room temperature for 5 minutes, and then added to the medium. 12 hours later, the shRNA-containing medium was replaced with fresh medium. 24 hours later, puromycin (1 μg/ml) was added to the medium for selection of transfected cells. All transfected cells were pooled 8 days later and maintained as a stable cell line. 1 million transfected RAW264.7 cells (shGHS-R or shScrambled) were seeded per well of 6-well plate, treated with LPS (1 μg/ml) for 16 hours, followed by harvest.

### Statistical analyses

We used repeated ANONA and two-tailed Student's *t*-test to determine statistical significance between genotypes or treatments. We expressed the results as: mean ± SEM. Statistical significance was set as *p* < 0.05.
